# Prediction of Neoadjuvant Chemoradiotherapy Response in Rectal Cancer with Metric Learning Using Pretreatment 18F-Fluorodeoxyglucose Positron Emission Tomography

**DOI:** 10.3390/cancers13246350

**Published:** 2021-12-17

**Authors:** Kuo-Chen Wu, Shang-Wen Chen, Te-Chun Hsieh, Kuo-Yang Yen, Kin-Man Law, Yu-Chieh Kuo, Ruey-Feng Chang, Chia-Hung Kao

**Affiliations:** 1 Graduate Institute of Biomedical Electronics and Bioinformatics, National Taiwan University, Taipei 106, Taiwan; d09945002@ntu.edu.tw; 2 Center of Augmented Intelligence in Healthcare, China Medical University Hospital, Taichung 404, Taiwan; D4634@mail.cmuh.org.tw (S.-W.C.); T35269@mail.cmuh.org.tw (K.-M.L.); A83020@mail.cmuh.org.tw (Y.-C.K.); 3 School of Medicine, College of Medicine, China Medical University, Taichung 404, Taiwan; 4 School of Medicine, College of Medicine, Taipei Medical University, Taipei 110, Taiwan; 5 Department of Radiation Oncology, China Medical University Hospital, Taichung 404, Taiwan; 6 Department of Nuclear Medicine and PET Center, China Medical University Hospital, Taichung 404, Taiwan; d10119@mail.cmuh.org.tw (T.-C.H.); T10540@mail.cmuh.org.tw (K.-Y.Y.); 7 Department of Biomedical Imaging and Radiological Science, China Medical University, Taichung 404, Taiwan; 8 Department of Computer Science and Engineering, National Chung Hsing University, Taichung 402, Taiwan; 9 Department of Computer Science and Information Engineering, National Taiwan University, Taipei 106, Taiwan; 10 Graduate Institute of Biomedical Sciences, School of Medicine, College of Medicine, China Medical University, Taichung 404, Taiwan; 11 Department of Bioinformatics and Medical Engineering, Asia University, Taichung 413, Taiwan

**Keywords:** 18F-fluorodeoxyglucose positron emission tomography/computed tomography, rectal cancer, neoadjuvant chemoradiotherapy, metric learning

## Abstract

**Simple Summary:**

Neoadjuvant chemoradiotherapy (NCRT) before surgery is the mainstay of treatment for patients with locally advanced rectal cancer. Based on baseline 18F-fluorodeoxyglucose ([18F]-FDG)-positron emission tomography (PET)/computed tomography (CT), a new artificial intelligence model was introduced to predict responses to NCRT. The model employed metric learning combined with the Uniform Manifold Approximation and Projection for dimensionality reduction. The treatment response was scored by Dworak tumor regression grade (TRG); TRG3 and TRG4 indicated favorable responses. Using this model, the area under the receiver operating characteristic curve was 0.96 for predicting a favorable response. The sensitivity, specificity, and accuracy were 98.3%, 96.5%, and 97.5%, respectively. After further external validation, oncologists may use the proposed model to advise patients on the relative suitability of treatment options, including the therapeutic decision between NCRT and neoadjuvant chemotherapy. Integrating this approach would have a notable effect on counseling patients about treatment alternatives or prognoses.

**Abstract:**

Objectives: Neoadjuvant chemoradiotherapy (NCRT) followed by surgery is the mainstay of treatment for patients with locally advanced rectal cancer. Based on baseline 18F-fluorodeoxyglucose ([18F]-FDG)-positron emission tomography (PET)/computed tomography (CT), a new artificial intelligence model using metric learning (ML) was introduced to predict responses to NCRT. Patients and Methods: This study used the data of 236 patients with newly diagnosed rectal cancer; the data of 202 and 34 patients were for training and validation, respectively. All patients received pretreatment [18F]FDG-PET/CT, NCRT, and surgery. The treatment response was scored by Dworak tumor regression grade (TRG); TRG3 and TRG4 indicated favorable responses. The model employed ML combined with the Uniform Manifold Approximation and Projection for dimensionality reduction. A receiver operating characteristic (ROC) curve analysis was performed to assess the model’s predictive performance. Results: In the training cohort, 115 patients (57%) achieved TRG3 or TRG4 responses. The area under the ROC curve was 0.96 for the prediction of a favorable response. The sensitivity, specificity, and accuracy were 98.3%, 96.5%, and 97.5%, respectively. The sensitivity, specificity, and accuracy for the validation cohort were 95.0%, 100%, and 98.8%, respectively. Conclusions: The new ML model presented herein was used to determined that baseline 18F[FDG]-PET/CT images could predict a favorable response to NCRT in patients with rectal cancer. External validation is required to verify the model’s predictive value.

## 1. Introduction

Neoadjuvant chemoradiotherapy (NCRT) before total mesorectal excision (TME) has become a mainstay of treatment for patients with locally advanced rectal carcinoma [1,2]. Responses to NCRT vary, with 15–27% of patients exhibiting a pathological complete response, 54–75% of patients exhibiting a partial response, and others exhibiting no response [3]. A phase three trial demonstrated that neoadjuvant chemotherapy with intravenous fluorouracil, leucovorin, and oxaliplatin without radiation achieved noninferiority in three-year disease-free survival relative to fluorouracil with radiation [4]. Determining whether a patient can achieve a favorable therapeutic response is crucial for counseling them on their treatment options and their decision on whether to undergo NCRT or neoadjuvant chemotherapy. The prediction of tumor responses before selecting NCRT maximizes the therapeutic benefits of the approach.

Among the imaging modalities used for clinical staging in patients with rectal cancer, 18F-fluorodeoxyglucose ([18F]FDG)-positron emission tomography (PET)/computed tomography (CT) imaging has been widely employed to assess patients’ pathological responses to NCRT [5,6,7,8,9]. The use of FDG-PET-derived radiomics for predicting favorable responses has also been investigated [10,11]. Artificial intelligence (AI) allows for novel image analysis techniques and may be key to the advancement of precision medicine. The authors of this study previously investigated the performance of a combination of baseline [18F]FDG-PET/CT radiomics and random forests in predicting pathological complete response in the same patient setting [12]. Compared with human-engineered radiomic methods, which strongly depend on segmentation methods and quantification of extracted features, a deep learning (DL) algorithm works by learning relevant features directly from image databases. Little is known regarding predictive performance when baseline [18F]FDG-PET/CT images are used in the absence of handcrafted features. Imaging features in [18F]FDG-PET/CT was hypothesized to be capable of directly predicting responses to NCRT using potential imaging biomarkers. In this study, a novel metric learning (ML) model with a data processing strategy was employed to circumvent the limitations of training on a cohort with a low data volume [13,14].

## 2. Methods

### 2.1. Study Design and Patient Population

**Figure 1:**
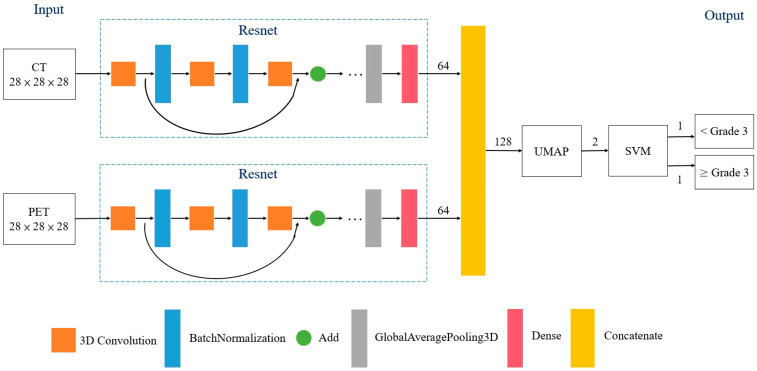
Structure of proposed model for classifying favorable responses to neoadjuvant chemoradiotherapy in patients with rectal cancer. Triplet loss for Resnet was utilized as a loss function for the ML algorithms. Abbreviations: UMAP = Uniform Manifold Approximation and Projection; SVM = support vector machine.

Between January 2009 and July 2018, 361 patients were screened for this retrospective study. They were newly diagnosed with rectal cancer and were scheduled to undergo curative NCRT followed by TME at our institute. All patients had biopsy-confirmed adenocarcinoma and received pretreatment [18F]FDG-PET/CT. No patients with mucinous or signet ring carcinomas were included. To minimize bias, patients who received TME more than 12 weeks after receiving NCRT were excluded. The structure of the proposed model for the classification of responses to NCRT is illustrated in Figure 1. The model was categorized as a supervised ML. The PET and CT images were processed and convoluted with ML separately. After two sets of the features were concatenated, dimensionality reduction was performed using a Uniform Manifold Approximation and Projection (UMAP). The treatment responses were classified using a support vector machine (SVM) according to the distribution of the visualized features. A receiver operating characteristic (ROC) curve analysis was performed to calculate the classification performance. This study was approved by China Medical University and Hospital Research Ethics Committee [certificate numbers: DMR99-IRB-010(CR-11) and CMUH106-REC3-119(CR-3)].

### 2.2. NCRT

The drugs used in the NCRT regimens comprised capecitabine, uracil-tegafur, and intravenous 5-fluorouracil. All patients were irradiated with intensity-modulated radiotherapy to reduce treatment-related toxicity without compromising the response rates [15]. After a prescribed dose of 45 Gy to the pelvis in 25 fractions over five weeks, a dose of 5.4 Gy in three fractions was administered as a boost to the gross tumor and metastatic pelvic lymph nodes.

### 2.3. Pathological Assessment

After the patients underwent TME, their pathological responses were scored according to the Dworak tumor regression grade (TRG) [16]. TRG3 or TRG4 responses were regarded as favorable, whereas TRG0, TRG1, and TRG2 responses were regarded as nonfavorable.

### 2.4. PET/CT Image Acquisition

The patients underwent [18F]FDG-PET/CT for baseline staging before NCRT. Before imaging, the patients fasted for at least 4 h to reduce the effect of serum glucose [12]. Approximately 60 min after 370 MBq of [18F]FDG was administered to the patients, images were taken using a PET/CT scanner (Discovery STE 16-Slice PET/CT Scanner, GE Healthcare, Milwaukee, WI, USA). The patients were required to rest during the uptake period. A CT topogram was used to label the axial scan range. After the CT was performed, PET images were obtained in the three-dimensional acquisition mode at 2 min per field of view (FOV) with an 11-slice overlap at the borders of the FOV. The CT performed was a low-dose non-contrast CT.

The [18F]FDG-PET data were saved in Advantage Workstation (Ver. 4.4, GE Healthcare). Two nuclear medicine physicians reviewed the images and located the target lesions. The PET/CT workstation quantified [18F]FDG uptake automatically.

### 2.5. Data Pre-Processing

The initial CT and PET images were reconstructed on a 512 × 512 and 128 × 128 matrix. To fit the size of the corresponding PET images, the matrix of CT images was converted to 128 voxels × 128 voxels × length of the region of interest (ROI). The geographical center of the tumors and the ROI of the lesions were defined based on the CT images. Through this approach, the training model converged more efficiently in the classification of responses to NCRT.

### 2.6. Metric Learning

ML is an AI method based on a distance metric that determines similarity or dissimilarity between objects [13,14,17]. This approach can decrease and increase the distance between similar and dissimilar objects, respectively. In this study, two deep residual learning frameworks were used to analyze PET and CT images, respectively. The batch normalization and activation of the rectified linear unit were performed before each block to minimize the possibility of overfitting (Figure 1). Furthermore, triplet loss was utilized as a loss function for the ML algorithms. The distances from the baseline input to the positive and negative inputs were therefore minimized and maximized, respectively. Consequently, the data were transformed into a new representation to facilitate classification training.

### 2.7. Uniform Manifold Approximation and Projection for Dimensionality Reduction

Dimensionality reduction plays a key role in data science. UMAP is a nonlinear dimensionality reduction technique that can be used for various data distributions through a combination of Riemannian geometry and algebraic topology [18,19]. UMAP has already been widely implemented in bioinformatics, materials science, and machine learning [18]. To improve visualization in the training model, a UMAP was used to reduce the dimensionality of the data by mapping it from high-dimensional to two-dimensional space. Through the use of this approach, the possibility of data overfitting or oversensitivity was minimized [20].

### 2.8. Support Vector Machine

An SVM is a machine learning algorithm that can efficiently engage in linear or nonlinear classification. In addition, SVMs are capable of categorizing low-volume data sets. This study utilized an SVM to classify preprocessed two-dimensional visualized features into two groups, namely, responses of below TRG3 and those of TRG3 or above.

### 2.9. Statistical Analysis

An ROC curve analysis was performed to calculate the classification performance. The area under the ROC curve (AUC) was used to evaluate the predictive performance of the model. The predictive indices included sensitivity (SE), specificity (SP), positive predictive value (PPV), negative predictive value (NPV), and accuracy. The analysis was performed using a commercial software (SPSS Statistics 26.00).

## 3. Results

### 3.1. Patient Characteristics

According to the patients’ treatment periods, their images, and available clinical data, the 236 patients were included in this study and they were divided into two cohorts (202 and 34 patients in the training and validation cohorts, respectively), as indicated in Appendix A. The patients in the training cohort were treated at any period between January 2009 and June 2017, whereas the patients in the validation cohort were treated on July 2017 or later. The same PET/CT scanner and treatment scheme were used during the patient inclusion. In the training cohort, the patients’ tumors were mainly located in the upper or rectosigmoid junction (16 patients), the middle third (103 patients), or the lower third (83 patients), as summarized in Table 1. The median age was 58 years (31–86 years); 140 patients were men, and 63 were women. The median interval from the end of NCRT to the TME was 56 days. In four patients with metastatic liver tumors before NCRT, wedge resection of liver tumors were carried out simultaneously during the TME. In total, 117 patients (58%) achieved TRG3 or TRG4 responses.

### 3.2. Partitioning of Patients in the Training Cohort

To ensure that training was not conducted on a fixed data set alone, this study applied K-fold cross-validation to validate the strength of the model. The patients were randomly divided into five groups, each containing a comparable proportion of TRG3 and TRG4 responders. Each set was labeled as a test set only once, and the remaining sets were combined to construct the training set for the modeling. During the training process, all sets included in the training cohort were split into training and test sets at a ratio of 4:1.

### 3.3. Image-Based Prediction

**Figure 2:**
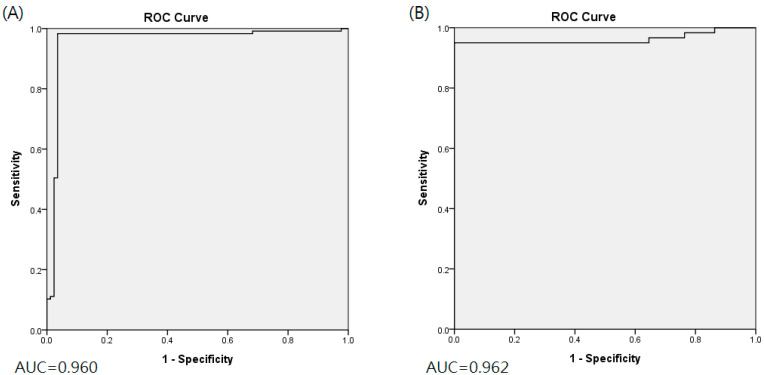
Area under ROC curve for training cohort (**A**) and validation cohort (**B**).

**Figure 3:**
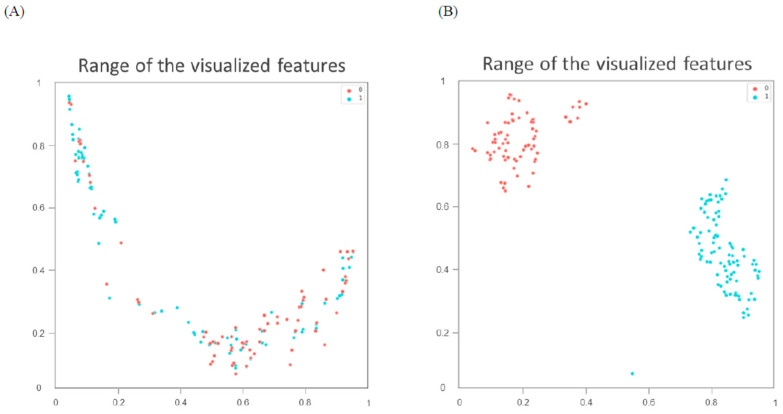
Distribution of visualized features for training model before (**A**) and after (**B**) metric learning and dimensionality reduction using Uniform Manifold Approximation and Projection. Blue spots represent tumor regression grade 3 or 4 responses; red spots correspond to responses lower than grade.

**Figure 4:**
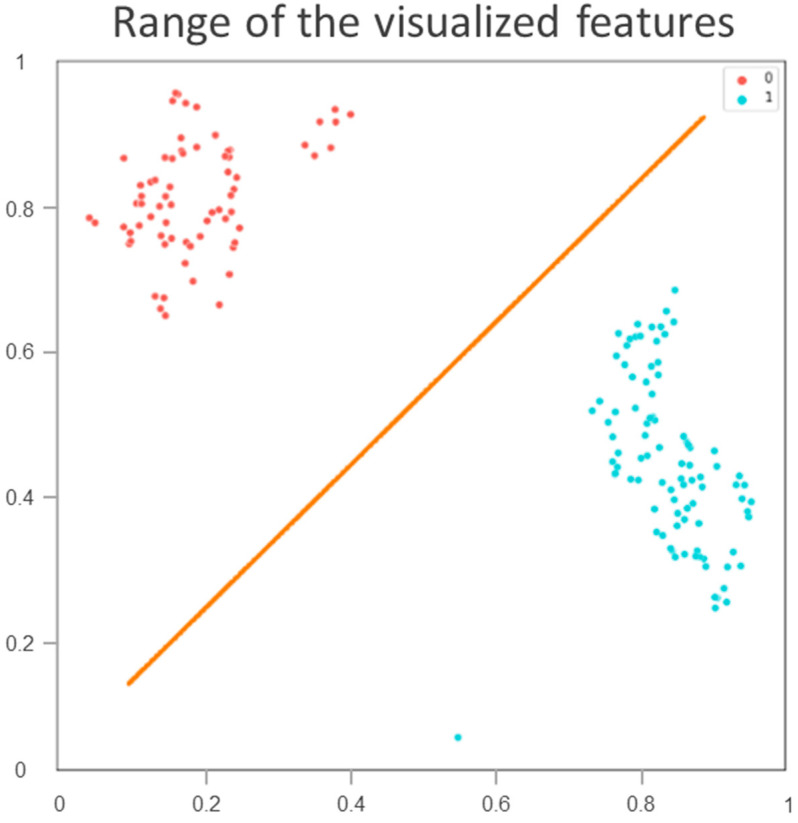
Classification performance of training cohort by support vector machine for tumor regression grade 3 or 4 responses following dimensionality reduction of visualized features. Blue spots represent tumor regression grade 3 or 4 responses; red spots correspond to responses lower than grade 3.

The classification indices for the prediction of TRG3 or TRG4 responses for all tumors in the five sets of the training cohort are summarized in Table 2. The AUC was 0.96 [95% confidence interval (CI) 0.951–0.993] (Figure 2A), and the predictive SE, SP, PPV, NPV, and accuracy were 98.3% (95% CI 0.962–1.000), 96.5% (95% CI 0.936–0.993), 97.5% (95%CI 0.958–0.993), 97.6% (95% CI 0.953–1.000), and 97.5% (95% CI 0.960–0.991), respectively. Figure 3 illustrates the distribution of the visualized features for the training model before and after the implementation of ML and UMAP, respectively. Figure 4 displays the overall classification performance of SVM for TRG3 and TRG4 responses, following the dimensionality reduction of the features. The proposed model could provide enhanced discrimination of the visualized two-dimensional features associated with a patient’s response to NCRT.

### 3.4. Validation and Comparison

The proposed model had the following predictive performance when applied to the PET/CT images of the 34 patients in the validation cohort. The AUC was 0.962 (95% CI 0.935–0.999) (Figure 2B), and the SE, SP, and accuracy were 95.0% (95% CI 0.910–0.990), 100% (95%CI 1.000–1.000), and 98.2% (95%CI 0.969–0.997), respectively. The classification performance of the proposed model and that of the traditional DL approach are compared in Appendix B. The AUC value of the DL without the integration of ML or UMAP was 0.618, which was significantly inferior to that of the proposed model (*p* = 0.002).

### 3.5. Heat Map

**Figure 5:**
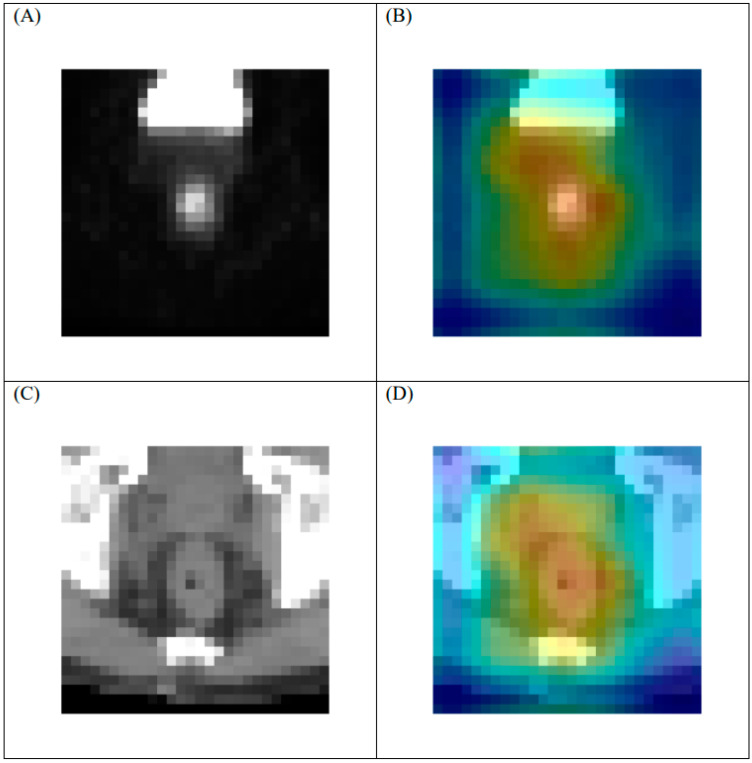
Representative heat map of tumor with regression grade 3 response on [F18]FDG-PET/CT in last layer of proposed model. (**A**) original PET, (**B**) heat map of PET, (**C**) original CT, and (**D**) heat map of CT. Note: 1. Heat maps were generated using a commercial software (Grad-CAM). 2. The geographical center of the PET and CT images is the same.

A heat map was utilized to visually identify the discriminative regions targeted by the proposed model and to detect events in the imaging set. The rectum is adjacent to many organs, including the uterus, bladder, and prostate. These anatomical structures, which also may exhibit an increased uptake of FDG, might disrupt the visualization and cause inaccuracies. Therefore, the heat map indicated the activated area in the imaging sets in the last layer of the ML model (Figure 5). The heat map demonstrated that the proposed model was capable of distinguishing the rectum from the adjacent organs. In addition, the characteristics of the target events were based on critical areas in the rectal tumors.

## 4. Discussion

Precision medicine for cancer treatment involves the identification of biological or imaging markers to predict therapeutic outcomes early. A patient’s response to NCRT is crucial because it directly affects their prognosis [3]. In addition, patients with rectal cancer—especially those with low-lying tumors—who exhibit a favorable response to NCRT could benefit from sphincter-saving procedures [1]. Furthermore, a response prediction is valuable in determining a patient’s therapeutic decision between NCRT and neoadjuvant chemotherapy. By employing a novel combination of ML and UMAP, this study demonstrated that baseline [18F]FDG-PET imaging could be used to classify a patient’s NCRT response with high accuracy. Although the gold standard for measurement of a tumoral response to NCRT is postoperative histopathological analysis, this proposed model can provide an innovative platform for future studies related to individualized treatments.

Despite the lack of a universal algorithm for extracting radiomic features from [18F]FDG-PET imaging of rectal cancer, Bang et al. [11]. investigated a set of radiomic features in 74 patients with rectal cancer. The authors reported that the kurtosis of the absolute gradient was related to tumor recurrence. However, no significant associations existed between radiomics and TRG. Lovinfosse et al. [10]. conducted a study involving 66 patients and discovered that total lesion glycolysis of a tumor is a significant predictor of a TRG3 or TRG4 response to NCRT. The aforementioned predictive value of FDG-PET-based radiomics for NCRT might imply that the imaging features of rectal tumors in FDG-PET are associated with a particular phenotypic response to NCRT. Although the biological mechanism underlying the imaging of tumor heterogeneity remains unclear, implementation of AI-based models may enable oncologists to identify a particular tumor phenotype common to patients predicted to respond favorably to NCRT.

Two studies have demonstrated that DL combined with features derived from magnetic resonance imaging (MRI) before NCRT exhibits superior performance in the prediction of patients’ treatment responses [21,22]. In a prospective study by Zhang et al., [21] 383 participants (290 in the training cohort, 93 in the test cohort) were evaluated using a DL model based on diffusion kurtosis MRI. The AUC was 0.99 for the prediction of TRG4 responses among participants in the test cohort but 0.79 for the prediction of downstaging of the primary tumors. Fu et al. conducted a study involving a cohort of 43 patients receiving NCRT and TME [22]. All of the patients underwent pretreatment diffusion-weighted imaging (DWI). The researchers found that DL of the features extracted from the DWI achieved significantly better classification of NCRT responses than that derived from handcrafted features. Therefore, to maximize predictive accuracy, future studies should integrate multiple sources of imaging information into the proposed model.

To achieve an accurate assessment of the proposed model’s clinical utility, this study employed an innovative AI method combining ML and UMAP to facilitate the measurement of training performance. DL does not always work well when training is conducted on a low-volume data set. In addition, the use of the algorithms to process the data might be time-consuming. DL and ML have been combined in deep metric learning [14]. This model is mainly based on the principle of similarity or connection between samples. Using this approach, the data can be transformed into a new feature space with a highly discriminative power. As indicated in Figure 3, UMAP can be implemented to reorganize the layout of the data distribution in a low-dimensional space to reduce the cross-entropy between the original and the low-dimensional topological representations [18]. Consequently, the features of treatment responses were effectively discriminated by the SVM according to the two-dimensional distribution. In the future, the performance of this proposed AI approach should be examined using other cancers or image settings to verify its reproducibility.

This study had several limitations. First, although the performance of the proposed model was validated using a validation cohort, validation with independent external data sets is still necessary to establish the model’s clinical utility because this study was conducted at a single institute. To optimize the role of the imaging phenotype, a model able to accurately predict the TRG4 response would be more beneficial. However, because a positive correlation was observed between predictive value and predicted events and because patients exhibiting TRG responses were a minority, the use of the proposed model to assess TRG4 responses might be challenging. Therefore, the authors of this study intend to increase the sample size to extend the predictive range of the proposed model.

Moreover, the overall predictive performance of the model can be strengthened by integrating information from other predictive models [21,22,23,24]. For example, the integration of MRI-derived features extracted before or after NCRT would be valuable because MRI-derived features are potentially associated with specific phenotypic categories observed in DWI and dynamic contrast-enhanced imaging [25]. Finally, future research should investigate disease-free or overall survival of patients to maximize the prognostic benefits of the imaging phenotypes. Nonetheless, this study’s findings represent a crucial step toward enabling customization of neoadjuvant therapy for patients with rectal cancer using AI. After further validation, oncologists may use the proposed model to advise patients on the relative suitability of treatment options, including the therapeutic decision between NCRT and neoadjuvant chemotherapy. Integrating this approach would have a notable effect on counseling patients about treatment alternatives or prognoses.

## 5. Conclusions

Using a novel ML model, this study demonstrated that baseline [18F]FDG-PET/CT images could be used to directly predict favorable responses in patients with rectal cancer who had received NCRT. Prior to its clinical application in personalizing patients’ treatment options, the proposed model requires further validation with more extensive clinical studies.

## Figures and Tables

**Table 1:** Patient characteristics of training cohort (*n* = 202).

Characteristic	Value
Age (years)	31–86 (median, 58)
Gender	Male:139, Female:63
Primary lesion location	
low rectum	83
middle rectum	103
upper rectum or rectosigmoid junction	16
CEA (ng/dL)	17.08 ± 37.92(0.48–241.88)
Pretreatment clinical staging (AJCC 7th ed.)	
T stage	T2:26, T3:148, T4:28
N stage	N0:60, N1:80; N2:62
M stage	M0:198, M1:4
Differentiation	
W-D	5
M-D	39
P-D	4
unknown	154
Concurrent chemotherapy regimen (%)	
Capecitabine	174
Uracil-Tegafur	21
Intravenous 5-Fluorouracil based regimen	7
Interval from the end of radiation to surgery	
>4 and <8 week	102
≥8 and <12 week	100
Tumor regression grade (%)	
Grade 0	0
Grade 1	31
Grade 2	54
Grade 3	93
Grade 4	24

Abbreviations: JCC = American Joint Committee on Cancer; CEA = carcinoembryonic antigen; W-D = well differentiated; M-D = moderately differentiated; P-D = poorly differentiated.

**Table 2:** Classification indices for tumor regression grade 3 or 4 responses in all five sets of the training cohort, comparing patients with and without favorable responses.

Prediction	RG Grade 3 or 4 Response		Indices
	Positive	Negative	
Positive	115	2	98.3%
Negative	3	82	96.5%
Indices	97.5%	97.6%	97.5%

**Figure A1:**
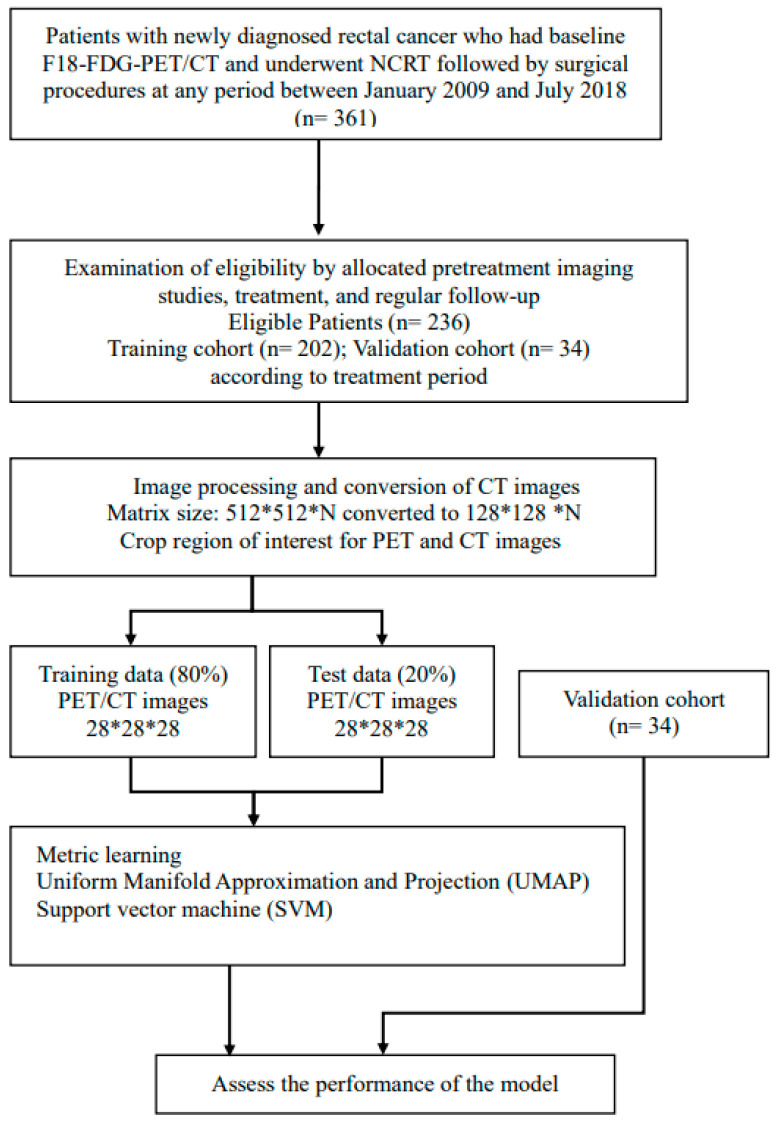
Flowchart of study design.

## Data Availability

The data reported results can be found with a link of https://drive.google.com/drive/folders/1lOYa-gQo-CyOdRFfeGcZSIQSc8bjDgzd?usp=sharing, accessed on 8 November 2021. The imaging raw data is too huge to be uploaded. Please contact with K.-C.W. if researchers are interested in this database.

## Abbreviations

NCRT: Neoadjuvant chemoradiotherapy; ML: metric learning; [18F]FDG-PET/CT: 18F-fluorodeoxyglucose-positron emission tomography/computed tomography; TRG: tumor regression grade; ROC: receiver operating characteristic.

## Appendix B

Comparison of classification performance between proposed model and traditional deep learning approach.

**Table A1:** Classification indices with metric learning and Uniform Manifold Approximation and Projection (UMAP).

	AUC (95% CI)		Sensitivity (95% CI)		Specificity (95% CI)		Accuracy (95% CI)	
Training cohort (*n* = 202)	0.960 (0.951–0.993)		0.983 (0.962–1.000)		0.965 (0.936–0.993)		0.975 (0.960–0.991)	
Validation cohort (*n* = 34)	0.962 (0.935–0.999)		0.950 (0.910–0.990)		1.000 (1.000–1.000)		0.982 (0.969–0.997)	
	**Fold_1**	**Fold_2**		**Fold_3**	**Fold_4**	**Fold_5**		**Summation**
Training AUC	0.993	0.944		1.000	0.964	0.959		0.972
Validation AUC	1.000	0.92		0.939	0.977	1.000		0.967
Training accuracy	0.976	0.976		1.000	0.950	0.975		0.975
Validation accuracy	1.000	0.971		0.971	0.971	1.000		0.982
Training sensitivity	1.000	1.000		1.000	0.957	0.957		0.983
Validation sensitivity	1.000	0.917		0.917	0.917	1.000		0.950
Training specificity	0.941	0.941		1.000	0.941	1.000		0.965
Validation specificity	1.000	1.000		1.000	1.000	1.000		1.000

Abbreviation: 95% CI = 95% confidence interval.

**Table A2:** Classification indices with traditional deep learning (ResNet) without UMAP or metric leaning.

	AUC (95% CI)		Sensitivity (95% CI)		Specificity (95% CI)		Accuracy (95% CI)	
Training cohort (n = 202)	0.618 (0.576–0.704)		0.630 (0.449–0.810)		0.588 (0.349–0.827)		0.614 (0.591–0.636)	
Validation cohort (n = 34)	0.606 (0.511–0.704)		0.467 (0.238–0.695)		0.745 (0.645–0.846)		0.647 (0.611–0.683)	
	**Fold_1**	**Fold_2**		**Fold_3**	**Fold_4**	**Fold_5**		**Summation SUM**
Training AUC	0.645	0.588		0.573	0.760	0.634		0.640
Validation AUC	0.542	0.614		0.716	0.458	0.708		0.608
Training accuracy	0.634	0.634		0.575	0.625	0.600		0.614
Validation accuracy	0.618	0.618		0.706	0.618	0.676		0.647
Training sensitivity	0.625	0.958		0.565	0.391	0.609		0.630
Validation sensitivity	0.333	0.583		0.583	0.083	0.750		0.467
Training specificity	0.647	0.176		0.588	0.941	0.588		0.588
Validation specificity	0.773	0.636		0.773	0.909	0.636		0.745

**Table A3:** Comparison of classification performance using statistical analysis. AUC value of traditional deep learning (ResNet) was 0.618, which was significantly inferior to that of the proposed model (*p* = 0.002).

	Number	Correlation	*p*-Value
Training cohort	202	0.215	0.002
Test cohort	34	0.308	0.076

## References

[1] Sauer R., Becker H., Hohenberger W., Rodel C., Wittekind C., Fietkau R., Martus P., Tschmelitsch J., Hager E., Hess C.F. (2004). Preoperative versus Postoperative Chemoradiotherapy for Rectal Cancer. N. Engl. J. Med..

[2] Bosset J.-F., Collette L., Calais G., Mineur L., Maingon P., Radosevic-Jelic L., Daban A., Bardet E., Beny A., Ollier J.-C. (2006). Chemotherapy with Preoperative Radiotherapy in Rectal Cancer. N. Engl. J. Med..

[3] Maas M., Nelemans P.J., Valentini V., Das P., Rödel C., Kuo L.-J., Calvo F.A., García-Aguilar J., Glynne-Jones R., Haustermans K. (2010). Long-term outcome in patients with a pathological complete response after chemoradiation for rectal cancer: A pooled analysis of individual patient data. Lancet Oncol..

[4] Deng Y., Chi P., Lan P., Wang L., Chen W., Cui L., Chen D., Cao J., Wei H., Peng X. (2019). Neoadjuvant Modified FOLFOX6 With or Without Radiation Versus Fluorouracil Plus Radiation for Locally Advanced Rectal Cancer: Final Results of the Chinese FOWARC Trial. J. Clin. Oncol..

[5] Calvo F.A., Domper M., Matute R., Martínez-Lázaro R., Arranz J.A., Desco M., Álvarez E., Carreras J.L. (2004). 18F-FDG positron emission tomography staging and restaging in rectal cancer treated with preoperative chemoradiation. Int. J. Radiat. Oncol..

[6] Capirci C., Rampin L., Erba P.A., Galeotti F., Crepaldi G., Banti E., Gava M., Fanti S., Mariani G., Muzzio P.C. (2007). Sequential FDG-PET/CT reliably predicts response of locally advanced rectal cancer to neo-adjuvant chemo-radiation therapy. Eur. J. Nucl. Med. Mol. Imaging.

[7] Cascini G.L., Avallone A., DelRio P., Guida C., Tatangelo F., Marone P., Aloj L., De Martinis F., Comella P., Parisi V. (2006). 18F-FDG PET is an early predictor of pathologic tumor response to preoperative radiochemotherapy in locally advanced rectal cancer. J. Nucl. Med..

[8] Guillem J.G., Moore H.G., Akhurst T., Klimstra D.S., Ruo L., Mazumdar M., Minsky B.D., Saltz L., Wong W.D., Larson S. (2004). Sequential preoperative fluorodeoxyglucose-positron emission tomography assessment of response to preoperative chemoradiation: A means for determining long term outcomes of rectal cancer. J. Am. Coll. Surg..

[9] Konski A., Hoffman J., Sigurdson E., Haluszka O., Engstrom P., Cheng J.D., Cohen S.J., Watson J.C., Eisenberg D., McGarrity E. (2005). Can Molecular Imaging Predict Response to Preoperative Chemoradiation in Patients with Rectal Cancer? A Fox Chase Cancer Center Prospective Experience. Semin. Oncol..

[10] Lovinfosse P., Polus M., Van Daele D., Martinive P., Daenen F., Hatt M., Visvikis D., Koopmansch B., Lambert F., Coimbra C. (2018). FDG PET/CT radiomics for predicting the outcome of locally advanced rectal cancer. Eur. J. Nucl. Med. Mol. Imaging.

[11] Bang J.-I., Ha S., Kang S.-B., Lee K.-W., Lee H.S., Kim J.-S., Oh H.-K., Lee H.-Y., Kim S.E. (2016). Prediction of neoadjuvant radiation chemotherapy response and survival using pretreatment [18F]FDG PET/CT scans in locally advanced rectal cancer. Eur. J. Nucl. Med. Mol. Imaging.

[12] Shen W.-C., Chen S.-W., Wu K.-C., Lee P.-Y., Feng C.-L., Hsieh T.-C., Yen K.-Y., Kao C.-H. (2020). Predicting pathological complete response in rectal cancer after chemoradiotherapy with a random forest using 18F-fluorodeoxyglucose positron emission tomography and computed tomography radiomics. Ann. Transl. Med..

[13] Weinberger K.Q., Saul L.K. (2009). Distance metric learning for large margin nearest neighbor classification. J. Mach. Learn Res..

[14] Lu J., Hu J., Zhou J. (2017). Deep Metric Learning for Visual Understanding: An Overview of Recent Advances. IEEE Signal Process. Mag..

[15] Samuelian J.M., Callister M.D., Ashman J.B., Young-Fadok T.M., Borad M.J., Gunderson L.L. (2012). Reduced Acute Bowel Toxicity in Patients Treated with Intensity-Modulated Radiotherapy for Rectal Cancer. Int. J. Radiat. Oncol..

[16] Dworak O., Keilholz L., Hoffmann A. (1997). Pathological features of rectal cancer after preoperative radiochemotherapy. Int. J. Colorectal. Dis..

[17] Kaya M., Bilge H. (2019). Deep Metric Learning: A Survey. Symmetry.

[18] McInnes L., Healy J., Melville J. (2018). UMAP: Uniform Manifold Approximation and Projection for Dimension Reduction. arXiv.

[19] Becht E., McInnes L., Healy J., Dutertre C.-A., Kwok I.W.H., Ng L.G., Ginhoux F., Newell E.W. (2018). Dimensionality reduction for visualizing single-cell data using UMAP. Nat. Biotechnol..

[20] Roweis S.T., Saul L.K. (2000). Nonlinear Dimensionality Reduction by Locally Linear Embedding. Science.

[21] Zhang X.-Y., Wang L., Zhu H.-T., Li Z.-W., Ye M., Li X.-T., Shi Y.-J., Zhu H.-C., Sun Y.-S. (2020). Predicting Rectal Cancer Response to Neoadjuvant Chemoradiotherapy Using Deep Learning of Diffusion Kurtosis MRI. Radiology.

[22] Fu J., Zhong X., Li N., Van Dams R., Lewis J., Sung K., Raldow A.C., Jin J., Qi X.S. (2020). Deep learning-based radiomic features for improving neoadjuvant chemoradiation response prediction in locally advanced rectal cancer. Phys. Med. Biol..

[23] Bibault J.E., Giraud P., Housset M., Durdux C., Taieb J., Berger A., Coriat R., Chaussade S., Dousset B., Nordlinger B. (2018). Deep learning and radiomics predict complete response after neo-adjuvant chemoradiation for locally advanced rectal cancer. Sci. Rep..

[24] Horvat N., Veeraraghavan H., Khan M., Blazic I., Zheng J., Capanu M., Sala E., Garcia-Aguilar J., Gollub M.J., Petkovska I. (2018). MR Imaging of Rectal Cancer: Radiomics Analysis to Assess Treatment Response after Neoadjuvant Therapy. Radiology.

[25] Jethanandani A., Lin T.A., Volpe S., Elhalawani H., Mohamed A., Yang P., Fuller C.D. (2018). Exploring Applications of Radiomics in Magnetic Resonance Imaging of Head and Neck Cancer: A Systematic Review. Front. Oncol..

